# Predicting asthma-related crisis events using routine electronic healthcare data: a quantitative database analysis study

**DOI:** 10.3399/BJGP.2020.1042

**Published:** 2021-10-19

**Authors:** Michael Noble, Annie Burden, Susan Stirling, Allan B Clark, Stanley Musgrave, Mohammad A Alsallakh, David Price, Gwyneth A Davies, Hilary Pinnock, Martin Pond, Aziz Sheikh, Erika J Sims, Samantha Walker, Andrew M Wilson

**Affiliations:** Acle, Norfolk, UK.; Observational and Pragmatic Research Institute, Singapore.; Norwich Medical School, University of East Anglia, Norwich, UK.; Norwich Medical School, University of East Anglia, Norwich, UK.; Norwich Medical School, University of East Anglia, Norwich, UK.; Swansea University Medical School, Swansea, UK.; Observational & Pragmatic Research Institute, 883 North Bridge Road, #02-05, Southbank, Singapore.; Swansea University Medical School, Swansea, UK.; Asthma UK Centre for Applied Research, University of Edinburgh, Edinburgh, UK.; Norwich Medical School, University of East Anglia, Norwich, UK.; Asthma UK Centre for Applied Research, University of Edinburgh, Edinburgh, UK.; Norwich Medical School, University of East Anglia, Norwich, UK.; Asthma UK, London, UK.; Norwich Medical School, University of East Anglia, Norwich Research Park, Norwich, UK.

**Keywords:** algorithms, asthma, asthma attack, general practice, prediction, risk

## Abstract

**Background:**

There is no published algorithm predicting asthma crisis events (accident and emergency [A&E] attendance, hospitalisation, or death) using routinely available electronic health record (EHR) data.

**Aim:**

To develop an algorithm to identify individuals at high risk of an asthma crisis event.

**Design and setting:**

Database analysis from primary care EHRs of people with asthma across England and Scotland.

**Method:**

Multivariable logistic regression was applied to a dataset of 61 861 people with asthma from England and Scotland using the Clinical Practice Research Datalink. External validation was performed using the Secure Anonymised Information Linkage Databank of 174 240 patients from Wales. Outcomes were ≥1 hospitalisation (development dataset) and asthma-related hospitalisation, A&E attendance, or death (validation dataset) within a 12-month period.

**Results:**

Risk factors for asthma-related crisis events included previous hospitalisation, older age, underweight, smoking, and blood eosinophilia. The prediction algorithm had acceptable predictive ability with a receiver operating characteristic of 0.71 (95% confidence interval [CI] = 0.70 to 0.72) in the validation dataset. Using a cut-point based on the 7% of the population at greatest risk results in a positive predictive value of 5.7% (95% CI = 5.3% to 6.1%) and a negative predictive value of 98.9% (95% CI = 98.9% to 99.0%), with sensitivity of 28.5% (95% CI = 26.7% to 30.3%) and specificity of 93.3% (95% CI = 93.2% to 93.4%); those individuals had an event risk of 6.0% compared with 1.1% for the remaining population. In total, 18 people would need to be followed to identify one admission.

**Conclusion:**

This externally validated algorithm has acceptable predictive ability for identifying patients at high risk of asthma-related crisis events and excluding those not at high risk.

## INTRODUCTION

The challenge of reducing unplanned hospital admissions and avoidable deaths for common chronic conditions, such as asthma, remains unresolved. Despite effective treatments, evidence-based guidelines,^1^ and financially incentivised community-based chronic disease management (via the Quality and Outcomes Framework^2^), each year in the UK an average of 1500 people die^3^ (on average, 3 a day) and 93 000 are hospitalised due to asthma.^4^ A total of 5.4 million people in the UK are currently receiving treatment for asthma: 1.1 million children (1 in 11) and 4.3 million adults (1 in 12).^3^ Identification of those at increased risk of these events is beneficial both at an individual level to tailor disease management, and at a population level to inform and modify processes of care.

Many risk factors for poor asthma outcomes have been identified,^5^^–^^8^ some of which have been combined into risk algorithms, including: Asthma UK’s Asthma Attack Risk Checker tool;^9^ the Asthma Disease Activity Score;^10^ and wheeze frequency, admissions, reliever use, and step on British Thoracic Society medication guidelines (WARS) score.^11^ Recently, an algorithm has also been developed to identify children at risk of life-threatening asthma.^12^ These have been derived from small datasets, including those from clinical trials, or the variables used in the prediction tools have required up-to-date personal characteristics, including psychosocial characteristics or adherence to medication for which comprehensive data are difficult to obtain in large populations.^13^ An algorithm to identify patients at greatest risk of poor outcomes using electronic healthcare data would overcome this problem and enable a register of patients at high risk to be generated efficiently.

Most prediction algorithms define a severe asthma attack as one that requires oral corticosteroid therapy or hospital attendance/admission;^14^ however, this composite scoring includes variables that are not necessarily colinear. Early treatment with prednisolone may stop the deterioration and prevent an accident and emergency (A&E) attendance and, as such, this composite definition may mask the benefits of prompt management of an attack, with increased prednisolone treatment and reduced hospitalisations;^13^ as such, it is important to develop algorithms that identify these two risks separately.

**Table:** How this fits in

Risk stratification is commonly undertaken in primary care but there are no validated prediction algorithms for people with asthma using routine data. An algorithm was developed using a primary care dataset and externally validated showing acceptable predictive ability with a receiver operating characteristic of 0.71 (95% confidence interval = 0.70 to 0.72). The 7% of the population most at risk had an event rate of 6.0%, compared with 1.1% for the remaining population. This algorithm can be used to identify individuals at high risk of an asthma-related crisis event from primary care electronic health records.

The authors aimed to develop and validate a prediction tool to identify individuals at high risk of an asthma-related crisis event (A&E attendance, hospital admission, or death due to asthma) during the following 12 months, calculated from routinely captured electronic health record (EHR) data.

## METHOD

### Data sources

#### Derivation dataset

An analytical dataset was used from a published cohort study^15^ that used a database of people aged 12–80 years registered at one of 650 primary care practices in the UK with physician-diagnosed and recorded asthma (with no subsequent code for asthma resolved), measurement of full blood count (FBC) at any time in the past, and 2 years’ continuous data. The dataset comprised data from the Clinical Practice Research Datalink (CPRD)^16^ between 2001 and 2012. Although the CPRD database contains record-linked primary and secondary care data, including reason for admission to hospital, only data from primary care were used to derive the algorithm because EHRs in UK primary care do not consistently code secondary care events. However, both primary and secondary care data were used when assessing the outcome.

#### Validation dataset

A separate dataset of patients from the Secure Anonymised Information Linkage (SAIL) Databank^17^^,^^18^ who were registered at 340 general practices in Wales was used to validate the algorithm. Record-linked data from primary and secondary care were available for individual patients and included reason for admission to hospital. Data on asthma outcomes, healthcare interactions (including GP consultations), and prescribed medications were obtained from the SAIL Databank.

### Eligibility

Patients included in the existing analytical dataset for the derivation of the at-risk algorithm comprised those with:

- active asthma (that is, with a coded diagnosis of asthma and a prescription for asthma treatment in the previous 12 months^19^);
- no diagnosis of any other chronic respiratory disease;
- a valid blood eosinophil count (≤5000 blood eosinophils/microlitre [µL]); and
- complete data for the baseline and outcome years (the year prior to, and the year following, the last eosinophil count, respectively).

Patients included in the SAIL Databank validation dataset comprised those with at least one asthma diagnosis code before 31 December 2011, no ‘asthma resolved’ codes between 1 January 2010 and 31 December 2011, and at least one asthma prescription (bronchodilator, corticosteroid, or leukotriene receptor antagonist) code between 1 January 2010 and 31 December 2010. Patients were continuously registered at one general practice between 1 January 2010 and 31 December 2010 (baseline data-collection year) and continually registered (or died) between 1 January 2011 and 31 December 2011 (outcome year).

### Predictors

Details of all variables considered as potential predictors for the at-risk algorithm are shown in Supplementary Table S1. These included age, sex, smoking history, comorbidities, respiratory-related medication, healthcare contacts, and blood eosinophil count. For diagnostic variables (for example, ischaemic heart disease and diabetes), Read codes were queried any time up to the end of the baseline year (that is, 31 December 2010) from the validation and derivation databases. Similarly, for blood eosinophil count, body mass index (BMI), and smoking status, the most recent codes any time before 31 December 2010 were used. For the rest of the variables — prescriptions for asthma, allergic rhinitis, diabetes, anxiety and depression, paracetamol use (which is positively associated with asthma^20^), lower respiratory tract infection (LRTI) consultations, allergic rhinitis diagnosis — the codes were queried between 1 January 2010 and 31 December 2010.

### Outcome

For the development of the algorithm, the outcome was defined as ≥1 hospitalisation(s) within 12 months; for the validation of the algorithm, it was defined as a crisis event that comprised an asthma-related hospitalisation, A&E attendance, or death within a 12-month period.

### Statistical analysis

Univariate logistic regression models were used to identify baseline measures of disease severity, patient demographics, and comorbidities predictive of ≥1 future event(s). Variables showing an association (*P*<0.05) with an asthma exacerbation resulting in hospital admission in univariable analyses were entered into a multivariable model, which was reduced using backward elimination to produce a final list of predictors of hospital admission. No model updating was undertaken.

The final model was used to create at-risk scores, indicating the risk of an asthma-related crisis event for each patient in the dataset. To do this, coefficients for those factors present in each patient were summed, along with the intercept, to obtain the risk score (x), which is the logit of the probability of asthma-related attendance at A&E or hospital admission; the probability is given by e^x^/(1+e^x^). Internal validation was not investigated, as a separate dataset was used to perform external validation. The calibration slope coefficient was estimated by splitting the predicted risk into 10 groups, based on deciles and calculating the percentage of people in those with the outcome, estimating a linear regression model with the predicted risk group against the actual risk.

Discrimination (the ability to distinguish between those who do, and do not, experience the outcome) was assessed by calculating the receiver operating characteristic (ROC) for the risk scores. In addition, the specificity, sensitivity, positive predictive values (PPVs), and negative predictive values (NPVs) were calculated for five different at-risk cut-offs (top 1%, 2%, 5%, 7%, and 10%) for the risk scores for both the derivation and the validation datasets. The overall goodness of fit of the score was assessed by estimating the pseudo *R*^2^ from the logistic regression model. Assuming an asthma prevalence of 6%–7%, a 7% cut-off would, on average, identify the most at risk 42–49 individuals from a practice of 10 000 patients. A sensitivity analysis was undertaken for the validation cohort including only data related to hospitalisation.

## RESULTS

### Participants

**Figure 1.**
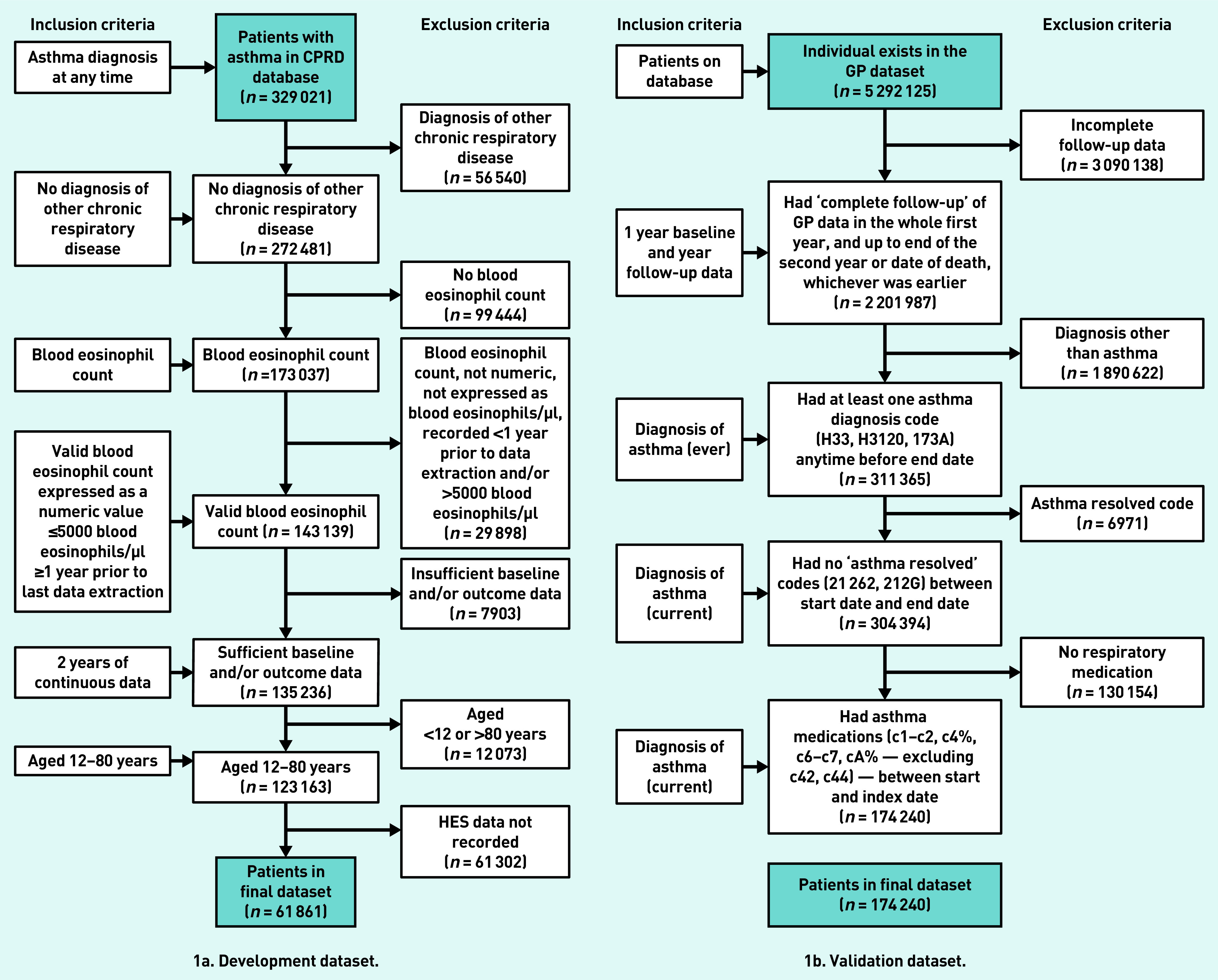
*Flow of participants through the study. CPRD = Clinical Practice Research Datalink. HES = Hospital Episode Statistics.*

The derivation and validation datasets comprised 58 619 and 174 240 people, respectively (Figure 1). The mean age of participants was 50 years in the derivation dataset and 44 years in the validation dataset, with more females than males in both datasets (Table 1). There were proportionally more people receiving Global Initiative for Asthma (GINA) treatment step 4 or 5 (medium- or high-dose inhaled corticosteroid and long-acting beta-agonist/muscarinic antagonist +/− add on therapies) and more with a diagnosis of, or treatment for, rhinitis in the derivation dataset than in the validation dataset (Table 1). There were differences between the datasets in terms of smoking status, BMI, anxiety and depression, and paracetamol usage. The outcome was present in 1.65% of individuals in the derivation dataset and 1.40% in the validation dataset (Table 1).

**Table 1.** Participant characteristics in the derivation (*n* = 58 619) and validation (*n* = 174 240) datasets^a^

**Characteristic**	**Derivation dataset, *n* (%)^b^**	**Validation dataset, *n* (%)^b^**
**Sex**		
Male	19 684 (33.58)	78 437 (45.02)
Female	38 935 (66.42)	95 803 (54.98)

**Age, years, mean (SD)**	49.70 (16.86)	44.47 (22.57)

**Outcome, derivation data: HES hospital admissions for asthma**		
≥1 admission	969 (1.65)	—
No admissions	57 650 (98.35)	—

**Outcome, validation data: asthma-related crisis event: hospital admission, A&E attendance, or death**		
Hospital admission	—	1434 (0.82)
A&E attendance	—	75 (0.04)
Death	—	1235 (0.7)
≥1 asthma events	—	2439 (1.40)^c^
No asthma events	—	171 801 (98.60)

**Age group, years**		
12– 60	40 809 (69.62)	125 802 (72.20)
61–80	17 810 (30.38)	48 438 (27.80)

**Smoking status**		
Current smoker	10 498 (17.91)	33 880 (19.44)
Ex-smoker	15 564 (26.55)^d^	70 544 (40.49)^e^

**BMI**		
Underweight:<18.5 kg/m^2^	1332 (2.27)	5160 (2.96)
Normal: 18.5–<25 kg/m^2^	18 403 (31.39)	82 221 (47.19)
Overweight: 25–<30 kg/m^2^	19 182 (32.72)	42 725 (24.52)
Obese: ≥30 kg/m^2^	19 702 (36.61)	44 134 (25.33)

**Count of blood eosinophils at baseline**		
≤400 µl	49 172 (83.88)	155 922 (89.49)
>400 µl	9447 (16.12)	18 318 (10.51)

**IHD**	3549 (6.05)	11 120 (6.30)

≥**1 LTRA prescriptions**	2871 (4.90)	11 258 (6.46)

**Diabetes diagnosis and/or therapy**	15 210 (25.95)	12 895 (7.40)

**Paracetamol**	18 482 (31.53)	28 018 (16.08)
**Rhinitis diagnosis and/or drugs in baseline**	27 845 (47.50)	27 127 (15.57)

**Number of courses of acute oral steroids**		
1	6444 (10.99)	17 390 (9.98)
2	2272 (3.88)	7083 (4.07)
≥3	2754 (4.70)	9056 (5.20)

**Previous hospitalisations**		
≥1	959 (1.64)	1057 (0.61)

**GP consultations for LRTI**		
1	7346 (12.53)	20 764 (11.92)
≥2	2550 (4.35)^f^	6990 (4.01)^g^

**Number of SABA prescriptions**		
0–2	30 368 (51.81)	73 688 (42.29)
3–6	17 912 (30.56)	51 725 (29.69)
7–12	7945 (13.55)	33 235 (19.07)
≥13	2394 (4.08)	15 592 (8.95)

**Anxiety and/or depression**	24 222 (41.32)	39 664 (22.76)

**History of anaphylaxis**	360 (0.61)	850 (0.49)

**GINA management steps, regrouped**		
No therapy	5180 (8.84)	519 (0.30)
Step 1–2	31 923 (54.46)	73 470 (42.17)
Step 3	7990 (13.63)	85 645 (49.15)
Step 4–5	13 526 (23.07)	14 606 (8.38)

a *This table contains frequencies of each variable for those individuals for whom all variables included in the algorithm were available.*

b *Unless otherwise stated.*

c *Individuals can have* ≥*1 event, so the total is less than the sum of the individual occurrences.*

d *Missing 32 557 were non-smokers.*

e *Missing 69 816 were non-smokers.*

f *The remaining 48 723 are ‘none’.*

g *The remaining 146 486 are ‘none’. A&E = accident and emergency. BMI = body mass index. GINA = Global Initiative for Asthma. HES = Hospital Episode Statistics. IHD = ischaemic heart disease. LRTA = leukotriene receptor antagonist. LTRI = lower respiratory tract infection. SABA = short-acting beta-agonist. SD = standard deviation.*

The results of the logistic regression are presented in Table 2, which gives the estimated weight of each variable and describes the algorithm used to predict asthma crisis events.

**Table 2.** Adjusted estimated regression coefficients in algorithm derivation

**Coefficient**	**β-coefficient (SE)**	***P*-value**
**Constant**	−5.013	—

**Factors considered for algorithm**		

**Age group, years, reference group** ≤**60 years**		
61–80	0.192 (0.076)	0.01

**BMI, kg/m^2^, reference group 18.5–**<**25 kg/m^2^**		
Underweight: <18.5 kg/m^2^	0.597 (0.179)	0.001
Overweight: 25– <30 kg/m^2^	−0.210 (0.087)	0.02
Obese: ≥30 kg/m^2^	−0.103 (0.083)	0.21

**Smoking status, reference group non-smoker**		
Current smoker	0.251 (0.089)	0.005
Ex-smoker	0.093 (0.078)	0.24

**Blood eosinophil counts, reference group** ≤**400/µl**		
>400/µl	0.237 (0.084)	0.005

**Rhinitis diagnosis and/or drugs**	−0.212 (0.068)	0.002

**Diabetes diagnosis and/or therapy**	0.378 (0.071)	<0.001

**IHD diagnosis**	0.243 (0.116)	0.036

**Anxiety and/or depression**	0.192 (0.068)	0.005

**History of anaphylaxis**	0.790 (0.275)	0.004

**GINA management step, reference group step 1–2**		
No therapy	0.568 (0.121)	<0.001
Step 3	0.101 (0.108)	0.35
Step 4–5	0.461 (0.078)	<0.001

**GP consultations for LRTIs**		
1	0.313 (0.086)	<0.001
≥2	0.206 (0.122)	0.09

**Acute oral steroids**		
1	0.551 (0.095)	<0.001
2	0.975 (0.120)	<0.001
≥3	1.141 (0.107)	<0.001

**Paracetamol**	0.204 (0.071)	0.004

**Hospitalisation, HES data**		
≥1	1.877 (0.105)	<0.001

*The pseudo R^2^ for the equation was 0.0846. Example: for a 24-year-old non-smoker with a BMI of 28 kg/m^2^, a history of rhinitis, and anxiety, who is receiving Step 3 GINA therapy and has had one course of prednisolone for asthma in the previous year, the at-risk probability = e^x^/(1 + e^x^) = e^−2.615^/(1 + e^−2.615^) = 0.01, where x = −4.591, calculated from −5.013 (constant) −0.210 (BMI) −0.212 (rhinitis) + 0.192 (anxiety) + 0.101 (asthma treatment) + 0.551 (asthma attacks). BMI = body mass index. GINA = Global Initiative for Asthma. HES = Hospital Episode Statistics. IHD = ischaemic heart disease. LTRI = lower respiratory tract infection. SE = standard error.*

The overall ability of the algorithm to discriminate between patients who subsequently had an asthma-related crisis event and those who did not was acceptable, and similar in the derivation dataset (ROC 0.72, 95% CI = 0.71 to 0.74) and the validation dataset (ROC 0.71, 95% CI = 0.70 to 0.72) (Table 3). Using a cut-point based on the 7% of the population at greatest risk results in a PPV of 5.7% (95% CI = 5.3% to 6.1%) and an NPV of 98.9% (95% CI = 98.9% to 99.0%), with sensitivity and specificity of 28.5% (95% CI = 26.7% to 30.3%) and 93.3% (95% CI = 93.2% to 93.4%), respectively (Table 3). The discriminative ability of the algorithm was similar in the validation cohort when the outcome was confined to hospitalisation only (see Supplementary Table S2). These individuals had a risk of event of 5.68% (Table 4) and 3.31% when considering hospitalisation only (see Supplementary Table S3). The at-risk algorithm showed acceptable prognostic performance in the validation data with a 5.4-fold higher asthma-related crisis event rate in the high-risk group (6.0%) versus the rest of the population (1.1%) at the 7% cut-off (Table 5) or an absolute difference of 4.9%.

**Table 3.** Measures of the prognostic performance of the algorithm in the derivation dataset (*n* = 58 619) and validation dataset (*n* = 174 240) (hospitalisation, death, or A&E attendance) for risk score thresholds of the top 1%, 2%, 5%, 7%, and 10% of patients with asthma

**Quintile**	**Derivation dataset, ROC 0.72 (95% CI**= **0.71 to 0.74)**					**Validation dataset, ROC 0.71 (95% CI**= **0.70 to 0.72)**				
	**Risk score threshold**	**PPV, %**	**NPV, %**	**Sensitivity, %**	**Specificity, %**	**Risk score threshold**	**PPV, %**	**NPV, %**	**Sensitivity, %**	**Specificity, %**
Top 1%	0.104	19.3	98.5	11.7	99.2	0.073	8.9	98.7	6.4	99.1
Top 2%	0.067	13.6	98.6	16.5	98.2	0.056	7.6	98.7	11.0	98.1
Top 5%	0.041	8.29	98.7	25.1	95.3	0.038	6.2	98.9	22.2	95.2
Top 7%	0.035	6.99	98.7	29.6	93.4	0.031	5.7	98.9	28.5	93.3
Top 10%	0.028	5.89	98.8	35.6	90.4	0.025	4.9	99.0	35.1	90.4

*A&E = accident and emergency. NPV = negative predictive value. PPV = positive predictive value. ROC = receiver operating characteristic.*

**Table 4.** Number of events by risk strata in the derivation dataset (*n* = 58 619) and validation dataset (*n* = 174 240) (hospitalisation, death, or A&E attendance) cohorts, with risk score thresholds of top 1%, 2%, 5%, 7%, and 10% of patients with asthma

**Quintile**	**Derivation dataset**			**Validation dataset**		
	**Risk score threshold**	**People, *n* (%)**	**Events, *n* (%)**	**Risk score threshold**	**People, *n* (%)**	**Events, *n* (%)**
Top 1%	0.104	587 (1)	113 (19.3)	0.073	1751 (1)	155 (8.85)
Top 2%	0.067	1173 (2)	160 (13.6)	0.056	3494 (2)	267 (7.64)
Top 5%	0.041	2931 (5)	243 (8.3)	0.038	8727 (5)	541 (6.20)
Top 7%	0.035	4106 (7)	287 (7)	0.031	12 225 (7)	694 (5.68)
Top 10%	0.028	5862 (10)	345 (5.9)	0.025	17 427 (10)	857 (4.92)
Total	n/a	58 619 (100)	969 (1.65)	n/a	174 240 (100)	2439 (1.40)

*A&E = accident and emergency; SAIL = Secure Anonymised Information Linkage.*

**Table 5.** Selected characteristics of individuals identified as being at risk, according to suggested cut-point of top 7%

	**Derivation dataset**		**Validation dataset**	

	**At risk, *n* (%)**	**Not at risk, *n* (%)**	**At risk, *n* (%)**	**Not at risk, *n* (%)**
**Total**	4106 (100.00)	54 513 (100.00)	10 042 (100.00)	164 198 (100.00)

**Age group, years**				
12–60	2250 (54.80)	38 559 (70.73)	3980 (39.63)	121 822 (74.19)
61–80	1856 (45.20)	15 954 (29.27)	6062 (60.37)	42 376 (25.81)

**Smoking status**				
Non-smoking	1879 (45.76)	30 678 (56.28)	1806 (17.98)	68 010 (41.42)
Current smoker	985 (23.99)	9513 (17.45)	2913 (29.01)	30 967 (18.86)
Ex-smoker	1242 (30.25)	14 322 (26.27)	5323 (53.01)	65 221 (39.72)

**IHD**				
No	3455 (84.15)	51 615 (94.68)	7868 (78.35)	155 252 (94.55)
Yes	651 (15.85)	2898 (5.32)	2174 (21.65)	8946 (5.45)

**History of anaphylaxis**				
No	3988 (97.13)	54 271 (99.56)	9797 (97.56)	163 593 (99.63)
Yes	118 (2.87)	242 (0.44)	245 (2.44)	605 (0.37)

**Diabetes and/or therapy**				
No	1957 (47.66)	41 452 (76.04)	7859 (78.26)	153 486 (93.48)
Yes	2149 (52.34)	13 061 (23.96)	2183 (21.74)	10 712 (6.52)

**Blood eosinophil count**				
≤400/µl	3113 (75.82)	46 059 (84.49)	8139 (81.0)	147 783 (90.0)
>400/µl	993 (24.18)	8454 (15.51)	1903 (19.0)	16 415 (10.0)

**BMI**				
Normal: 18.5– <25 kg/m^2^	1121 (27.3)	17 282 (31.7)	3943 (39.27)	78 278 (47.67)
Underweight: <18.5 kg/m^2^	221 (5.38)	1111 (2.04)	638 (6.35)	4522 (2.75)
Overweight: 25– <30 kg/m^2^	994 (24.21)	18 188 (33.36)	2094 (20.85)	40 631 (24.75)
Obese:≥29 kg/m^2^	1770 (43.11)	17 932 (32.89)	3367 (33.53)	40 767 (24.83)

**GP consults for LRTIs**				
0	1982 (48.27)	46 741 (85.74)	4014 (39.97)	142 472 (86.77)
1	1341 (32.66)	6005 (11.02)	3521 (35.06)	17 243 (10.50)
≥2	783 (19.07)	1767 (3.24)	2507 (24.97)	4483 (2.73)

**Rhinitis and/or therapy**				
No	2359 (57.45)	28 415 (52.13)	8616 (85.8)	138 497 (84.35)
Yes	1747 (42.55)	26 098 (47.87)	1426 (14.2)	25 701 (15.65)

**Anxiety and/or depression**				
No	1698 (41.35)	32 699 (59.98)	5534 (55.11)	129 042 (78.59)
Yes	2408 (58.65)	21 814 (40.02)	4508 (44.89)	35 156 (21.41)

**Acute oral steroids**				
0	643 (15.66)	46 706 (85.68)	543 (5.41)	140 168 (85.37)
1	817 (19.90)	5670 (10.40)	972 (9.68)	16 418 (10.0)
2	957 (23.31)	1336 (2.45)	2058 (20.49)	5025 (3.06)
≥3	1689 (41.13)	801 (1.47)	6469 (64.42)	2587 (1.58)

**Paracetamol**				
No	1729 (42.11)	38 408 (70.46)	6481 (64.54)	139 741 (85.11)
Yes	2377 (57.89)	16 105 (29.54)	3561 (35.46)	24 457 (14.89)

**GINA management step**				
No therapy	213 (5.19)	4967 (9.11)	17 (0.17)	502 (0.31)
Step 1–2	934 (22.75)	30 989 (56.85)	803 (8.0)	72 667 (44.26)
Step 3	389 (9.47)	7601 (13.94)	3404 (33.9)	82 241 (50.09)
Step 4–5	2570 (62.59)	10 956 (20.10)	5818 (57.94)	8788 (5.35)

**Baseline hospital admissions^a^**				
No	3158 (76.91)	54 502 (99.98)	8989 (89.51)	164 194 (100)
Yes	948 (23.09)	11 (0.02)	1053 (10.49)	<5 (0)

**Outcome: asthma-related crisis event^b^**				
No	3819 (93.01)	53 831 (98.75)	9438 (93.99)	162 363 (98.88)
Yes	287 (6.99)	682 (1.25)	604 (6.01)	1835 (1.12)

a *In the validation dataset, actual values are masked due to small frequencies in one category.*

b *Hospitalisation or A&E attendance in derivation dataset, and any of hospitalisation, A&E attendance, or death in validation data. A&E = accident and emergency; BMI = body mass index; GINA = Global Initiative for Asthma; IHD = ischaemic heart disease; LTRI = lower respiratory tract infection.*

The calibration slopes showed acceptable agreement between deciles of mean risk score and proportions of people experiencing asthma-related crisis events in each decile group, with data points close to the line of equality. The slope coefficient for the derivation dataset was 0.99 (95% CI = 0.92 to 1.05), while that for the validation was 0.85 (95% CI = 0.75 to 0.96) (data not shown).

## DISCUSSION

### Summary

Using data that are routinely available in UK primary care EHRs, the authors derived and externally validated an algorithm containing hospitalisation, older age, underweight, smoking, and blood eosinophilia variables to identify individuals at increased risk of experiencing an asthma-related crisis event. This had acceptable overall characteristics with an ROC of 0.72 in the derivation and 0.71 in the validation cohorts, respectively. Using the top 7% of the score as a cut-off, the algorithm correctly identified 28.5% of the asthma population most at risk and 93.3% of those not at risk. A practice can expect a crisis event to occur in 6.0% of the group that is at risk compared with 1.1% of the rest of the population with asthma. Eighteen people would need to be followed to identify one admission. The algorithm can identify people who are at a five-fold increased risk (absolute difference of 5%) of an asthma-related crisis event compared with those not at risk.

### Strengths and limitations

The main strength of this study is that it used two separate large databases capturing people from different geographical areas with record linkage between primary and secondary care data. The generalisability of the algorithm is illustrated by its similar behaviour in two different datasets. The data on cause (asthma related or not) for hospital admission when deriving the algorithm were deliberately ignored as this information, although predictive of future events, is not routinely available in primary care datasets. However, by linking primary care data with that from secondary care for the purposes of assessing the outcome, it was possible to confirm that the algorithm identifies people at risk of an asthma-related crisis event.

The limitations were that patients in the derivation, but not the validation, cohort needed to have had a valid FBC to be entered into the database (although specific values, such as eosinophil counts, were not required). This may have resulted in differences in some of the characteristics (for example, age, sex, asthma severity, and number of comorbidities); however, the authors do not believe there is any difference in the diagnosis or management of people with asthma between Wales and England as both countries follow national guidelines.^1^

The databases contained data that are now a decade old (validation 2001–2012, validation 2011–2012) and asthma guidelines have been updated in this time. Modifications made have included the use of high-dose inhaled corticosteroids to abort an asthma attack,^21^ vitamin D monitoring and therapy,^22^ and the use of monoclonal antibody therapies.^23^ However, there have been no marked changes to the understanding of the aetiology of asthma crises or deaths since the data were collected, and the software systems and determinants of coding decisions in day-to-day practice remain comparable. The authors did not, however, have access to information on medication adherence or social circumstances.

Socioeconomic status has been shown to be a risk factor for hospitalisation^24^ and an independent predictor for life-threatening asthma in children.^12^ Unfortunately, routine data do not contain this information, although algorithms have been developed for assessing prescription uptake^25^ and socioeconomic status is available from postcode data,^26^ both of which may be applied to future algorithms.

In addition, the authors did not have death or A&E data in the derivation cohort, although it was available for the validation cohort. Despite this, it has been shown that the performance of the prediction algorithm is similar when considering both hospitalisation alone or the composite of hospitalisation, A&E attendance, or death.

Although the number of short-acting beta-agonist (SABA) prescriptions were included in the list of potential variables, long-acting beta-agonist as monotherapy (which has been described as a risk factor in asthma deaths^27^) was not, as this regime is rarely prescribed.^28^ This algorithm does not predict community-based asthma attacks requiring oral prednisolone.

### Comparison with existing literature

The WARS score had an ROC of 0.83 for prednisolone use,^11^ but the performance of the score in terms of crisis events is unknown; likewise, the performance measurements of the risk score developed by Bateman *et al*^10^ for asthma attacks are not published. However, the Respiratory Effectiveness Group initiative published an algorithm to predict the risk of ≥2 attacks in the subsequent 2 years with an ROC of 0.79 (95% CI = 0.78 to 0.79).^29^ Recent evidence^27^ suggests that disease severity is an unreliable measure of risk and, indeed, the results presented here confirmed that GINA treatment step ‘no therapy’ was as statistically significant a risk factor as steps 4–5.

In terms of non-respiratory hospitalisation prediction algorithms, the QRISK2 score — which is widely used in the NHS to predict cardiovascular events — has an *R* ^2^ of 43.5 and 38.4, and an ROC statistic of 0.82 and 0.79 for females and males, respectively.^30^ A systematic review of risk prediction models to predict emergency admission in community-dwelling adults^31^ identified 27 different such models and showed that those using clinical data (as in the algorithm presented here) outperformed those using self-reported data; C-statistics ranged from 0.63 to 0.83. The algorithm presented here, which utilised clinical data, had a comparable level of calibration (C-statistic 0.72) to other clinically useful algorithms. Outcome data were collected as events over a 12-month period to avoid seasonal variations. The algorithm, therefore, predicts hospitalisation in the following year; however, an individual’s risk status can change if, for example, they had a hospitalisation just within, or without, of a 365-day period. Different algorithms can show substantial variation in risk at the individual level^32^ and should complement physician assessment based on knowledge about individuals.

Nevertheless, the growing workloads on primary care clinicians and the ongoing challenge of rising unplanned admissions and avoidable deaths makes accurate identification and targeting of the individuals at highest risk an essential part of primary care strategy.

### Implications for practice

Primary care software systems routinely use prompts to alert clinicians to overdue asthma reviews and the overordering — and, by implication, overuse — of SABAs. Both are helpful markers of risk that are not always recognised as such,^13^^,^^33^^,^^34^ but they do not reflect the range and complexity of factors found in patients who are most at risk of adverse outcomes.^27^^,^^35^ Current guidelines recommend that patients are assessed for risk of future attacks. The indicators recommended include a history of previous attacks, SABA use, and other markers of disease control, atopy, and environmental tobacco exposure in children; in adults, these include smoking, obesity, and depression.

In April 2020, Quality and Outcomes Framework indicators for disease control were changed^36^ from ‘Royal College of Physicians (RCP) 3 questions’ (on asthma):^37^

*Have you had difficulty sleeping because of your asthma symptoms (including cough)? Have you had your usual asthma symptoms during the day (for example, cough, wheeze, chest tightness, or breathlessness? Has your asthma interfered with your usual activities (for example, housework, work, school)?*

to the Asthma Control Test score plus the number of exacerbations in the previous 12 months. Achieving these new indicators requires more clinician time and greater participation from patients. Failure to attend appointments is, in itself, a risk factor for poor outcomes.^35^

The algorithm developed and presented here simplifies the collection and weights the statistical significance of multiple risk factors. It has the potential to save clinicians’ time and provide accurate real-time assessments of patients’ risk and, as it does not require patients to attend a consultation, also bypasses the dangers of inverse care associated with poor attendance at appointments. The algorithm also concurs with, and provides a mechanism to identify, important markers highlighted in the National Review of Asthma Deaths report,^27^ such as patients on no treatment for their asthma. It can be used to generate alerts or prompts to identify patients at high risk of asthma crisis events (A&E attendance, hospitalisation, or death), when their EHRs are accessed so care can be targeted appropriately.

The algorithm is currently being used in a study^38^ to validate the role of at-risk asthma registers in primary care. Further work is also needed to explore some of the unexpected indicators, such as low BMI, and to find a way to incorporate important social and behavioural determinants that are not currently captured in primary care EHRs.
